# Annotations

**Published:** 1898-02-26

**Authors:** 


### ANNOTATIONS.

### Excess of Sanitary Zeal.

THERE seems no limit to the lengths to which some people will go in regard to sanitary legislation. According to the *Australasian Medical Gazette* a Bill has been introduced to the effect that it shall not be lawful for any person suffering from any form of tuberculosis to land in New Zealand, or for the master of any vessel arriving there to allow him to land, and that if any passenger is found to be suffering from any form of tuberculosis within three months of landing in the country he shall, until the contrary is proved, be deemed to have been suffering from that disease when he landed. Breaches of these regulations are to be punished by fines. Of course, this is all very pretty, but we should like to be told how a captain of a ship is to know whether a passenger is suffering from "any form" of tuberculosis, and how an unfortunate person who happens to develop tuberculosis within three months of arrival is to "prove" that he was *not* suffering from tuberculosis when he landed. This is legislation gone mad. When people are asked to pass laws against overcrowding, or adulteration, or dirt, or vice, or what not, they hesitate and throw all sorts of difficulties in the way. To pass a law against the admission of a disease, however, seems to them in their innocence as simple as A B C! As if everyone was ticketed with the names of all his maladies.

### The Convict on the Road.

"GIVE us the luxuries of life, and we will dispense with the necessaries," an American humorist once exclaimed, and the sentiment is carried out in practice by many of his countrymen. So, at least, it seems to a European, who notes everywhere a hundred conveniences, intended to make life easier and more agreeable, while such a primary necessity as good roads is almost neglected. There may be reasons for this neglect, of course. The American is always in such a hurry to go on and push his fortunes farther, that he never thinks of retracing his steps, and therefore cares little for the roads he leaves behind. He shakes the dust of them off his feet, though, if our recollection of American journeying is correct, he finds it almost impossible to brush it off his garments. It is possible, too, that if a community, a city, a state, should decide to improve its roads, the contract for the work would be given on political grounds to someone who would take the money, but would not consider it needful to do the work. Such things do happen in the Great Republic now and then. But we are glad to see that in North Carolina there is a determination to improve the roads, and that it is being done in a wise and successful fashion. Convicts are being employed in road-making, with the best results. Some of the results are, indeed, surprising. One is that it costs about threepence a day less to maintain a convict when he is working on the roads than when he is in prison. Another is that, being offered various inducements in the way of shortening their term of imprisonment and other rewards, the convicts work as faithfully and well as any free labourer. They have a foreman to direct their work, as any gang of labourers might, but no guard. It seems almost to bring imprisonment down to a sham when, as we are told, convicts are permitted to go home from Saturday to Monday; but, however contrary to our preconceived ideas of discipline this may be, it seems to answer well enough, for not a convict has taken advantage of this privilege to attempt escape. We on this side have long ago found that convict labour might be employed on public work without wronging any one; but in the States the intense jealousy of the "free labourer" has prevented the labour of the convict being put to any useful purpose. At the present day, if the inmates of Elmira Reformatory build a wall in order to learn that simple kind of work, they must afterwards knock it down. The consciousness that their work is absolutely useless will tend to make even convicts more languid in their efforts. But, as the *New York Evening Post* says, in alluding to the North Carolina experiment, "Such a use of convicts will not displace 'honest labour,' as in many States well-directed work on the roads is not now done at all."

### Ether Followed by Chloroform.

IN commenting on the lists of deaths from anæsthetics which we have published from time to time, we have remarked on the frequency with which chloroform syncope, or poisoning, as it should more properly be called has arisen in the early stage of administration. In a lecture recently delivered at the London Hospital Dr. Hewitt made some very valuable remarks in regard to the plan of continuing anæsthesia by chloroform after having first induced it by ether. He pointed out, as Hill and others have done, that deaths taking place in the early stage are usually due primarily to rigidity, struggling, and "holding the breath"; secondarily, to a considerable quantity of the anæsthetic being taken in during the succeeding respirations, so that the heart becomes paralysed by the chloroform carried directly to it. But by giving ether and chloroform in succession the stage of rigidity and excitement, which is the dangerous stage under chloroform, is passed over under the stimulating effects of ether. Fatalities during this stage are practically unknown under ether. Having secured a proper degree of anæsthesia, chloroform may be substituted. Dr. Hewitt says that he has adopted this principle for several years, and that he finds that he is thus able to use chloroform without those untoward symptoms which are bound to occasionally arise when it is used from the commencement of administration. "Should the operation be a short one, and should the administration of ether be attended by no difficulties, little, if anything, is to be gained by changing to chloroform. But should ether cause cough, embarrassed breathing, or the secretion of much mucus, or should the operation be likely to be a protracted one, a change to chloroform is certainly advisable." Anæsthesia, however, should not be deep when the change is made, or too much chloroform may be absorbed by the rapid respiration and brisk circulation brought about by the ether. Generally speaking, the conjunctival reflex should be present when the change is effected. Administration may, if preferred, be commenced with nitrous oxide, but the stage of rigidity should be got over under ether. After ether anæsthesia has once been properly established, very little chloroform will be required to keep up the proper degree of unconsciousness. "Given that no special contra-indication exist, I believe," says Dr. Hewitt, "that there is no better plan of anæsthetising than this."

